# Daring to share requires intentional and collective commitment to civil discourse

**DOI:** 10.1186/s12966-020-00950-7

**Published:** 2020-04-07

**Authors:** Michael W. Beets, R. Glenn Weaver, Keith Brazendale

**Affiliations:** 1 Arnold School of Public Health, University of South Carolina, Columbia, SC USA; 2 Department of Health Sciences College of Health Professions and Sciences, University of Central Florida, Orlando, FL USA

## Abstract

Communicating and challenging ideas through written scholarly dialogue is a cornerstone of scientific progress. In the current social and political climate, it is important to reflect upon what constitutes appropriate ways to engage in scholarly dialogues and consider the ramifications of failing to create an environment where individuals are willing to share ideas openly. In this commentary, we provide examples of scholarly dialogues representing antagonistic and collegial tones and discuss the consequences of failing to communicate appropriately in the areas of gatekeeping, mentoring, and most importantly, the silencing of ideas.

Recently, through the act of writing and reading debates (i.e., commentaries), our group reflected on the process by which opposing viewpoints are expressed. We began to wonder what impressions readers were left with regarding the appropriateness of communicating alternative scientific perspectives when reading our work, and that of others.

Did we achieve what we believe to be the spirit of scholarly debates - advancing scientific ideas and challenging one another to think critically - or were we simply perpetuating entrenched ideas and/or dismissing the opposing ideas of others? We were unsure how readers and authors alike would know the difference between these two and whether scholarly debates, as a whole, model appropriate ways to engage in scientific dialogue.

In the current social and political climate where discourse among individuals with opposing views is often polarized and is characterized by personal attacks, it is paramount that such tactics are not widely adopted nor endorsed within the scientific community [1–3]. Should such conventions become commonplace within science, scientific progress could be stymied. Further, we wonder if existing practice in debates aligns with new initiatives from the International Society of Behavioral Nutrition and Physical Activity to encourage the sharing of ideas to advance science (#dare2share) and the Society of Behavioral Medicine invitation to members to share “big ideas” and “provocative questions” to address major challenges faced by behavioral scientists.

We thought it timely and important, therefore, to discuss what we believe constitutes appropriate ways to engage in scholarly dialogues and to consider the ramifications of failing to create an environment where individuals are willing to share ideas openly.

## Defining civil dialogue

Civil discourse has a long- and well-established history. From philosophers to nonprofit thinktanks and institutes, the topic of civil discourse and its importance for maintaining a healthy and productive society have been widely studied and debated. Civil discourse can take many forms, but is commonly characterized as language that has the following attributes [4, 5]: respect for others, productive, truthful, involves listening, avoids hostility and direct antagonism, and impersonality of reporting. Conversely, uncivil discourse is language characterized as containing direct insults, willful misattribution of motive without due reason, and open contempt [6].

In the sciences, the purpose of engaging in civil discourse is to find mutual benefit and opportunities for synergy amongst alternative perspectives for advancing a given field of study. It is not about entrenchment within scientific dogma or belittling one’s opponent simply because of disagreements. Being civil does not demand nor require a disconnect from passion. In fact, the majority of scientific scholars are intensely passionate about the topics they pursue. Passion fuels excitement, creativity, and strong feelings. Yet these should not serve to silence opposing viewpoints.

The following represent examples of both civil and uncivil discourse. These are not meant to be exhaustive nor will they be attributed to the original source in an effort to avoid singling out any individual. Rather, these are provided to illustrate how opposing views can be expressed that elicit either antagonistic or collegial tones.

*“…[my colleague’s] arguments… reflect an acute lack of understanding… and, as a consequence, should be summarily dismissed.”*

*“…[my colleague’s] arguments… barely warrant rebuttal.”*

*“…it was clear that [my colleague] is actually clinging to a much more constrained definition…”*

*“…[my colleague’s] seemingly irrational constraint… is critical to his case…”*

*“…the authors appear to have ignored… as a factor in their analysis…”*

*“…[my colleague] is hitting a screw with a hammer…”*

*“…they deliberately minimized the contributions of… and appeared to ignore strategically important published literature…”*

*“…the well-articulated reflections… are a gratifying indication that the conversation has begun”*

*“…I find [my colleague’s] contribution… very useful. It forced me to rethink…”*

*“…despite the strength of their arguments, I am not yet convinced. First of all, I want to see evidence.”*

*“…the paper… presents a thought provoking contribution... I fully agree with their arguments… I also agree with their implicit argument, and the explicit arguments made by others… However, the practical implications… seem limited.”*

*“…we appreciate a scientific community that participates in the conversation and asks for clarification when it is needed…”*

*“…we thank [the author’s] for their comments and feedback on our article…”*

Differentiating civil from uncivil dialogue should be intuitive. Based on these select examples, it may be clear to readers which of the statements are categorized as such. In some instances statements are made that do not appear to contribute to advancing a dialogue, whereas other statements acknowledge differences of opinions, quality points of an argument, and provide constructive non-dismissive counterpoints. These examples clearly demonstrate that disagreements can (and should) occur but can be done so civilly.

## Practical implications

While these examples serve to illustrate ways in which we can engage in productive scholarly dialogue, the larger practical implications of increasing/reducing the civility shown to one another are many. Of these, we believe there exists three main reasons scientists should strive for civility.

### We’re all gatekeepers

At some point, each of us will serve in a professional capacity as a gate keeper. This could be in the form of peer-reviews of scientific manuscripts, reviewer of grant applications, organizer of national or international conferences, etc.… Each one of these activities is accompanied with a power differential that can be wielded to influence the communication of ideas or funding of important projects. Engaging in uncivil discourse in one setting could result in the perceptions of stonewalling in other professional areas, not because of differences in viewpoints but ones based upon personal attacks or agendas.

### Mentoring the next generation

A vast majority of scientists, by the very nature of their profession, are in positions where they routinely mentor the next generation of research scientists. From these relationships, mentees often learn how to interact within a given profession based upon the mentor’s behaviors. Demeaning a colleague or summarily dismissing alternative viewpoints, whether in written or spoken word, demonstrates these are acceptable ways in which differences of opinion are expressed. Given the immediacy with which written debates/commentaries can spread in the digital-age, not only are we role modeling for those in direct proximity, but for an entire scientific community. Thus, we need to be mindful that uncivil behavior may nurture incivility in subsequent generations.

### Silencing ideas

Perhaps the greatest impact uncivil dialogue can have is the silencing of ideas. Uncivil dialogue can create an environment where others, with less power or prestige, are afraid or hesitant to present alternative ideas for fear of retribution or scientific bullying or being outcasted. Thus, ideas that could be transformative never reach the scientific community simply because a culture where the rigidity of viewpoints reigns supreme and anything presented in opposition is unwelcome.

The intentional engagement in civil discourse does not mean that we cannot disagree. Disagreements are a cornerstone of scientific advancement and help to challenge the way we think and view the world. But we do need to be mindful about the way in which we express disagreements and whether we interject language that serves to belittle another rather than constructively further a conversation.

Encouraging civil discourse can take multiple forms but requires intentional effort to ensure adoption and consistent use. These include course(s) on this topic embedded within graduate degree programs, trainings for peer reviewers of grants and manuscripts, oversight by journal editors and editorial boards, and conference workshops. Perhaps the most important form is the role modeling each of us should exhibit throughout all our professional interactions. Along with this, it should be noted that, when others demonstrate uncivil discourse, we need to feel comfortable in politely, yet constructively, coaching them to identify ways to communicate the same idea without being adversarial.

We hope this commentary has been helpful in understanding what we can do as a field to create environments where everyone’s viewpoint is valued and has an opportunity to be heard without criticism or fear of backlash. In total, we’re all in this together and everyone is trying to do better than what we’ve done before – but this needs to be done with civility.

## Data Availability

NA
